# Early Outcomes of Lisfranc Injuries Treated with Arthrex InternalBrace: A Case Series

**DOI:** 10.1007/s43465-024-01097-4

**Published:** 2024-02-02

**Authors:** Meloria Hoskins, Patrick Wise, Alicia Unangst, Philip Shaheen, Christopher Kreulen, Michael Aynardi, Eric Giza

**Affiliations:** 1 Department of Orthopaedic Surgery, Penn State Milton S. Hershey Medical Center, Penn State Hershey Bone and Joint Institute, 30 Hope Dr Suite 2400, Hershey, PA 17033 USA; 2 Department of Orthopaedic Surgery, University of California Davis Medical Center, Academic Offices 4860 Y Street, Suite 3800, Sacramento, CA USA

**Keywords:** Tarsometatarsal, Intercuneiform instability, Flexible fixation, Return-to-work, Foot trauma, Ligamentous

## Abstract

**Introduction:**

The treatment of Lisfranc injuries continues to evolve with time. The purpose of this study was to report early outcomes of patients with Lisfranc ligamentous injuries treated with the Arthrex InternalBrace, which has benefits to other previously described techniques.

**Materials and methods:**

We retrospectively identified 15 adult patients with Lisfranc injuries that were treated via open reduction internal fixation with the Arthrex InternalBrace (Naples, Fl). These patients were identified at two separate United States institutions between 2019 and 2022. Demographic data, mechanism of injury, and concomitant foot injuries were recorded. Outcomes were assessed by return-to-work or sport and time to weight-bearing. Secondary complications or revision surgeries were noted.

**Results:**

The mean patient age was 35 years. Eight patients had isolated Lisfranc ligamentous injuries and seven had additional intercuneiform instability, which required a supplemental limb of the fixation device. The most common mechanism of injury was a cutting/pivoting maneuver (*n* = 5) followed by fall (*n* = 4). The mean radiographic follow-up time was 7.3 months. The average time to weight-bearing as tolerated was 6.6 weeks (± 2.2). The average time to return-to-work/sport as tolerated was 14.1 weeks (± 3.6). Only two minor complications were noted at follow-up but no major complications or revision surgeries occurred.

**Conclusions:**

The outcomes of this case series suggest that the Arthrex InternalBrace is a viable option when performing open reduction and internal fixation of Lisfranc ligamentous injuries. Future prospective studies are needed to directly compare this device with alternative fixation methods.

**Graphical Abstract:**

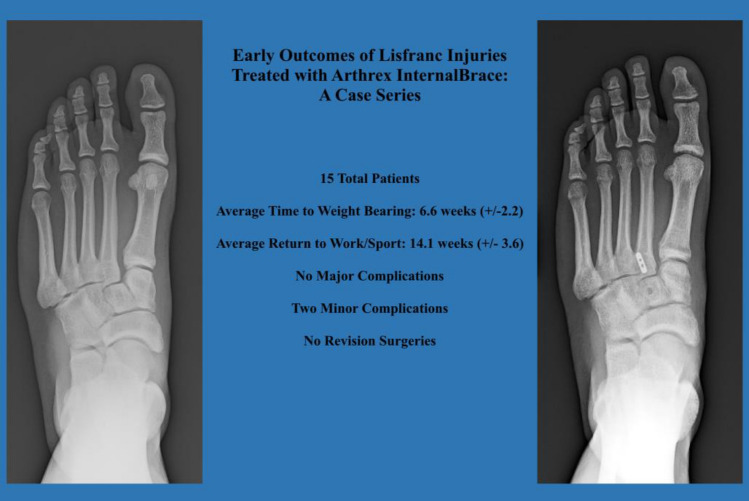

## Introduction

Lisfranc fractures account for approximately 0.20% of all fractures and are caused by a variety of injuries to the tarsometatarsal joint ranging from low-energy ligamentous sprains to high-energy motor vehicle accidents [1]. However, this injury is missed within nearly 20 to 30% of multi-trauma patients. As a result, the true incidence of these fractures may be underestimated [2]. Signs and symptoms of low-energy Lisfranc injuries may not be as obvious as high-energy injuries but can include severe pain, inability to bear weight, medial plantar bruising, swelling to the affected tarsometatarsal (TMT) joints [3]. Dorsal subluxation during application of a dorsal force to the forefoot while the other hand palpates the tarsometatarsal joints is suggestive of global instability [3]. Lisfranc injuries commonly are not isolated but have associated intercuneiform instability or base fractures.

The treatment of Lisfranc injuries has evolved over the past 20 to 30 years. Initial treatment options consisted of closed treatment with cast immobilization. Similar to most orthopedic injuries, this evolved to first percutaneous fixation and then open reduction internal fixations. In the late 1980s to 1990s, there was nearly universal acceptance of an open reduction and internal fixation method [4]. In many instances, this included the insertion of transarticular screws which created greater stability [5]. However, there have been reports of complications with the screws, which include damage to articular cartilage, irritation to the patient, loosening, and hardware failure [1]. Consequently, the screws must often be removed which can be difficult if the screws are fractured. Recently, there has been a trend toward suture button and dorsal bridge plate constructs to find techniques that will decrease disruption of the articular cartilage and potentially allow for more physiological movements [6, 7]. There are some studies which discuss the biological motion with flexible fixation but currently the specific use of InternalBrace is still being investigated, though incorporated into practice. The purpose of our paper was to retrospectively review those ligamentous Lisfranc injuries treated with InternalBrace fixation and see if they have similar time to weight-bearing, and return-to-work as previously published papers.

## Materials and Methods

Institutional Review Board approval was obtained prior to initiating study. We retrospectively identified all patients with Lisfranc joint injuries that were treated via open reduction internal fixation with the Lisfranc Fixation System for InternalBrace™ (Arthrex, Inc., Naples, FL) at two separate level 1 trauma academic institutions. Inclusion criteria for the study and use of the InternalBrace were: (1) patients that had clinical instability of the midfoot, (2) diastasis between second metatarsal base and the medial cunieform was at least 2 mm greater than contralateral side on weight-bearing films, (3) MRI-confirmed Lisfranc ligament tear or CT-confirmed simple Lisfranc ligamentous avulsion.

These patients were treated by three foot and ankle fellowship trained surgeons between May 2019 and August 2022. Informed consent was obtained prior to all procedures. We identified 15 total patients who underwent fixation using the InternalBrace system in this time period. The mean follow-up was 219 days (range: 43–563).

Demographic data, mechanism of injury, and concomitant foot injuries were recorded. Outcomes were assessed by return-to-work or sport and time to weight-bearing. Patients’ records were reviewed to evaluate for any known secondary complications or operations. Major complications were defined as return to operating room, surgical site infections requiring revision, loss or failure of reduction, revision procedures, and “hardware failure”, which were determined by increased diastasis of the Lisfranc interval on weight-bearing radiographs.

## Surgical Technique

**Fig. 1:**
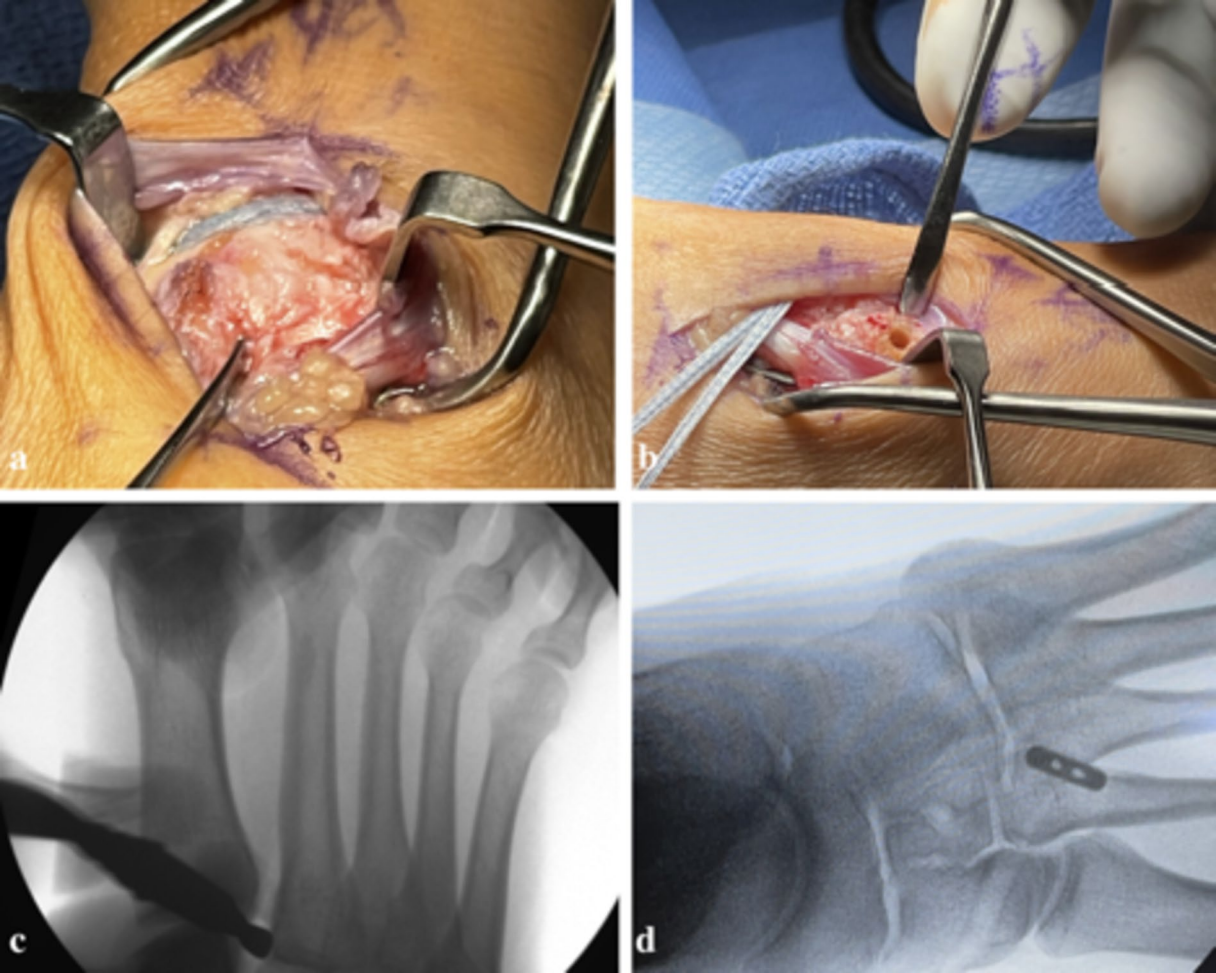
**a** Evaluating Lisfranc disruption through medial window; **b** K-wire insertion site at the dorsal lateral edge of the second metatarsal base; **c** intraoperative Freer elevator “drive through” sign; **d** final fluoroscopy image demonstrating correct placement of SwiveLock anchor [8]

Surgical operation is performed under general anesthesia with a preoperative or postoperative regional block. A dorsal incision is performed for both exposure and reduction via three separate windows. A medial window is utilized to evaluate the Lisfranc articulation with the intercuneiform joint (Fig. 1a), an intermediate window is created for vessel dissection and protection, and lateral window for exposure of the lateral aspect of the second metatarsal base and hardware implantation is used as well. Stress examination (pronation/abduction) and Freer elevator “drive-through” sign is performed to confirm a Lisfranc ligament tear (Fig. 1c) and evaluate for subtle disruption of intercuneiform joints when present.

**Fig. 2:**
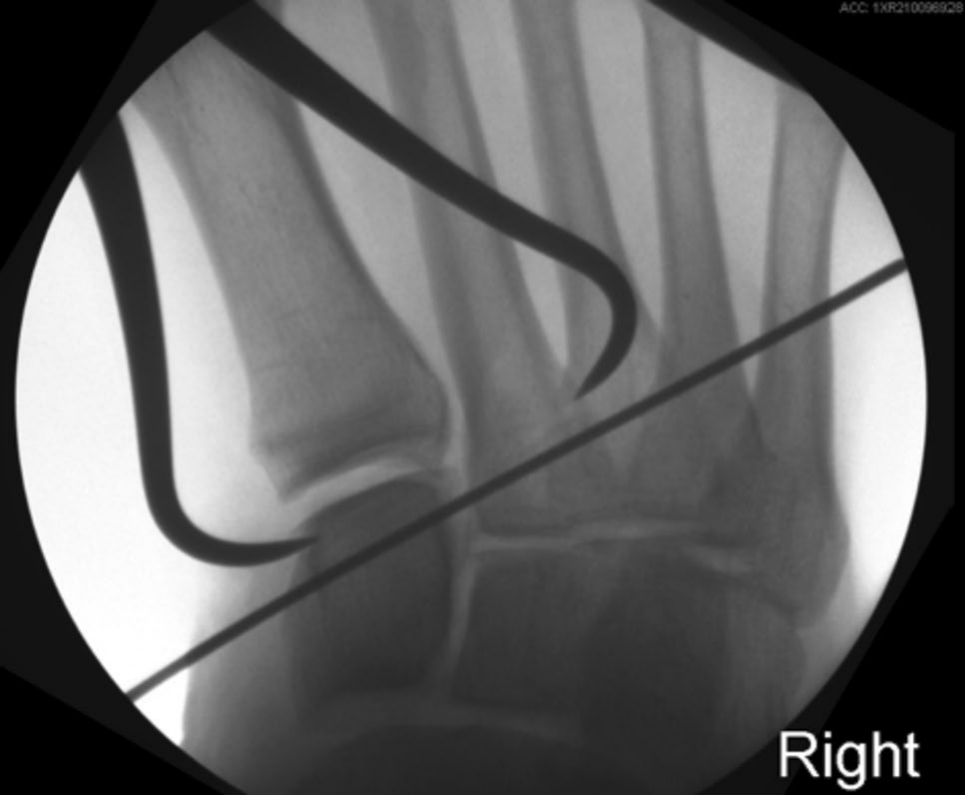
Weber clamp placement for reduction of Lisfranc joint

**Fig. 3:**
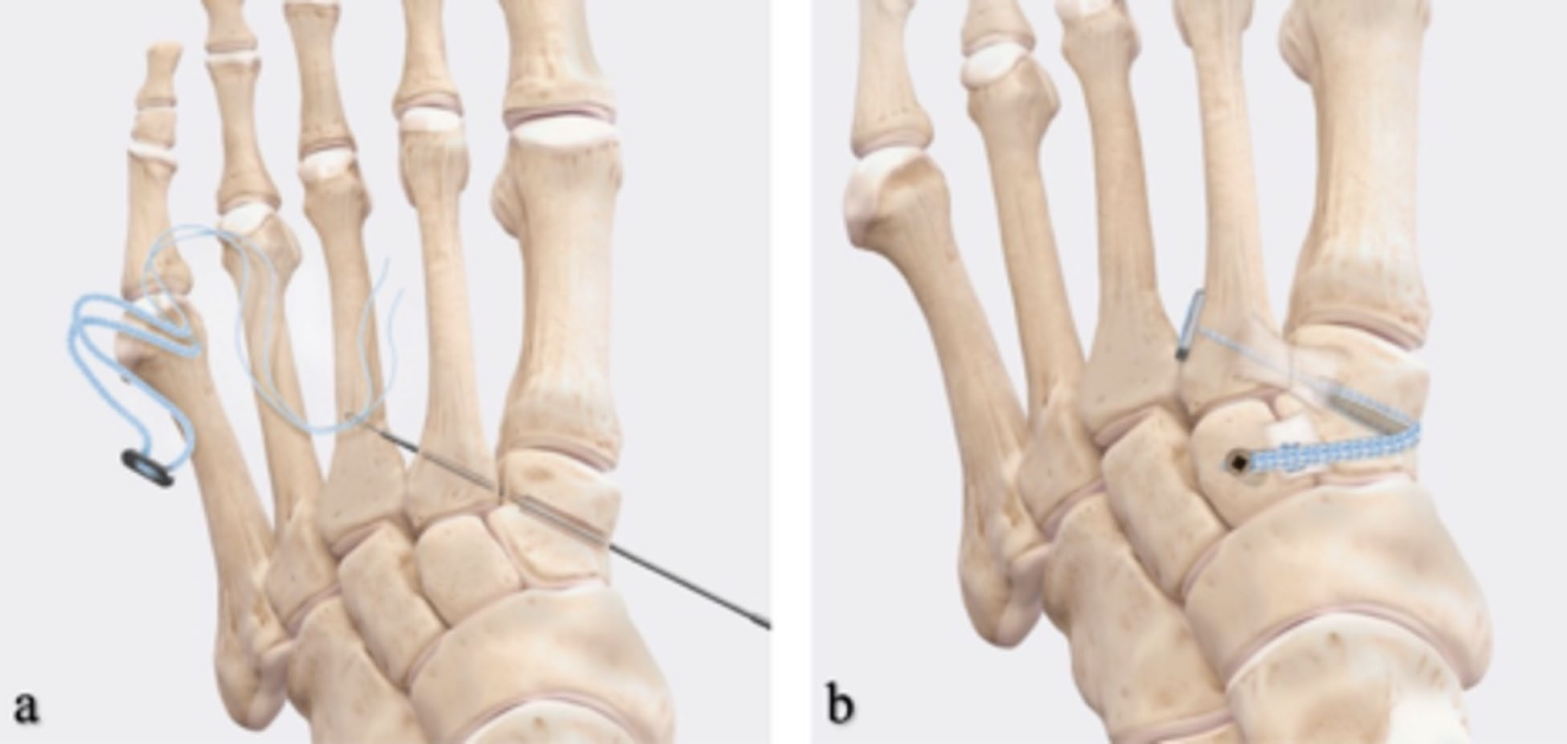
**a** Collagen-coated 3 mm FiberTape suture with the oblong button loaded through the 1.6 mm guidewire, **b** final fixation [8]

**Fig. 4:**
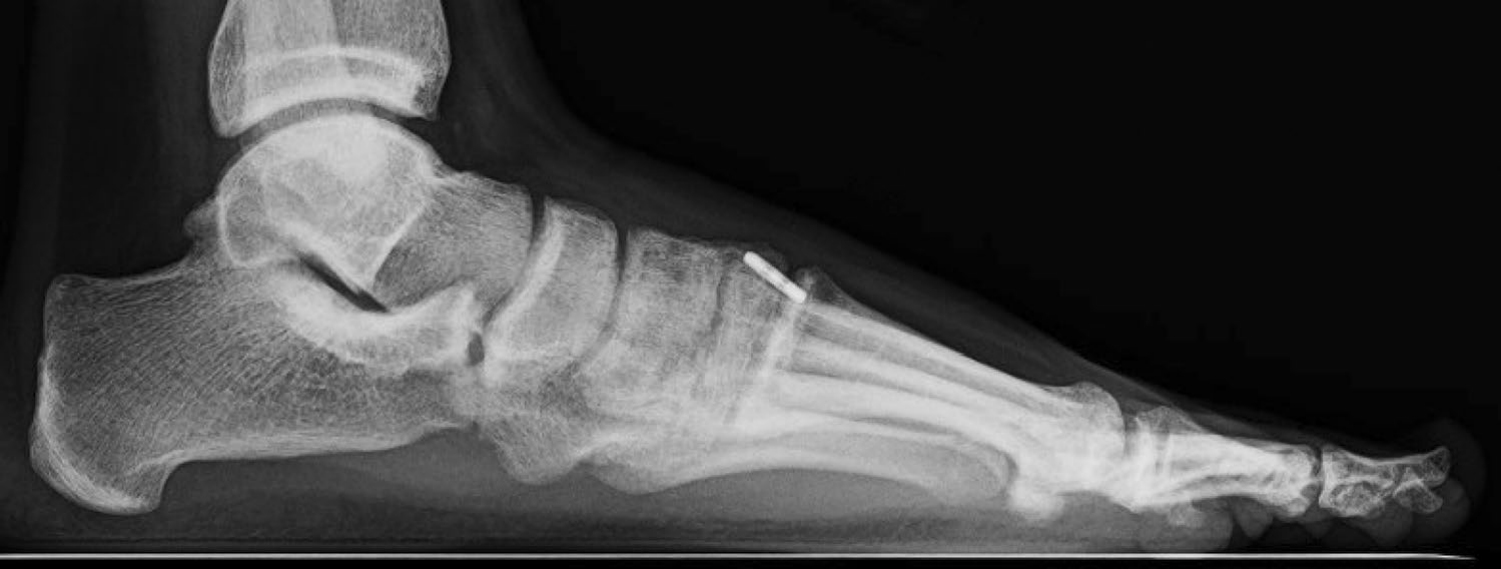
Postoperative lateral radiograph after Lisfranc IB application

The Lisfranc interval and intercuneiform space are debrided of fibrous tissue. Reduction is then obtained with a Weber clamp on the base of the second metatarsal and medial cuneiform through two small incisions, medial and dorsolateral (Fig. 2). Reduction is confirmed on AP view with less than 2 mm of diastasis and on lateral with no dorsal subluxation of second metatarsal. Guidewire is placed from the lateral base of the second metatarsal to the medial cuneiform (Fig. 3a). A collagen-coated 2 mm suture button and tape are passed from lateral to medial, with the button laying flush against the second metatarsal cortex. A 4.75 mm SwiveLock anchor is placed in the medial cuneiform to hold the Lisfranc interval reduced after predrilling and tapping (Fig. 3b). Proper positioning of implant on a postoperative lateral radiograph can be seen in Fig. 4.

If intercuneiform instability is present, the tape limbs are brought subperiosteal to the dorsum of the foot, under all dorsal soft tissue structures, to the middle cuneiform where a second 3.5 mm interference anchor is placed seating the supplemental limb (Figs. 3b, 4).

## Results

Of the 15 patients identified, 8 were male and 7 were female. Ten out of fifteen patients were white and there was only one smoker in the group (Table 1). Average age at time of surgery was 35.2 with a range of 18–66 years old. Eight of the fifteen had purely ligamentous injuries and seven had ligamentous plus intercuneiform instability and other metatarsal base fractures. Five of the injuries were sustained during cutting/pivoting type maneuvers, three occurred from inversion, four patients fell on a plantarflexed foot, and three were due to crush injuries (Table 2). We had an average follow-up time of 7.3 months (1.5–18.8) (Table 3). There was no sign of “hardware failure” in any patients through follow-up. There were two documented minor complications but no major complications. One patient had a suspected medial bursitis near the medial interference screw and one had continued hypersensitivity of the foot greater than 6 weeks postoperatively (Table 3). Our patients had full return to non-protected weight-bearing at 6.6 weeks (4.1–13.1). They had documented return-to-work at an average of 14.1 weeks (11.6–24.0) (Table 3).

**Table 1:** Patient demographics

Patient characteristics	*n* = 15
Mean age	35.2
Age range	18–66
Female	7
Male	8
Athletes	7
Race/ethnicity	
White, non-Hispanic	10
Non-White, non-Hispanic	5
Smokers	1

**Table 2:** Lisfranc injury types and mechanisms

	*n* = 15
Injury type	
Ligamentous injury	8
Ligamentous injury compounded with intercuneiform instability	7
Mechanism of injury	
Crush	3
Fall	4
Inversion	3
Cutting maneuver	5

**Table 3:** Outcome measures

Outcome	*n*	SD	Range
Follow-up time (months)	7.3 (*n* = 15)	5.5	1.4–18.8
Average time to weight-bearing as tolerated (weeks)	6.6 (*n* = 15)	2.2	4.1–13.1
Average time to return-to-work/play as tolerated (weeks)	14.1 (*n* = 13)	3.6	11.6–24.0
Complications	*n* = 15		
Surgical site infection	0		
Hypersensitivity	1		
Revisions	0		
Medial bursitis	1		
6-week postoperative X-ray	*n* = 15		
Hardware complications	0		
12-week postoperative X-ray	*n* = 14		
Hardware complications	0		

## Discussion

Ligamentous Lisfranc injuries are debilitating injuries and can lead to morbidity and disability if missed or under treated. Unfortunately there is no consensus in the current literature for the best way to treat these injuries. Treatment options vary from dorsal bridge plating, endobuttons, FiberTape suture anchors to primary fusions. Primary fusion has been shown to be effective in 92% of patients in getting those back to their pre-injury level; however, there can still be a need for secondary surgeries for screw removal and the larger soft tissue disruptions. Using any form of flexible “fibertape” fixation for ligamentous Lisfranc injuries can help alleviate the risk for hardware removal and potentially allow for subtle motion which may mimic that of the native joint. The InternalBrace (IB) FiberTape device (Arthrex, Naples, FL) specifically has potential for collagen ingrowth, potentially limits iatrogenic cartilage damage during insertion, and has been shown to withstand more cyclical loading than suture button fixation devices. Furthermore, its use eliminates the need for a button on the medial cuneiform that could irritate the tibialis anterior tendon [9, 10].

The use of the IB has been biomechanically studied in two separate studies. The first paper compared IB alone with IB with supplemental limbs across the intercuneiform joint in a cadaver model. The second was a cadaver study comparing the InternalBrace (IB) load to failure against screw fixation [3, 11]. In these publications, the investigators found that the load to failure was greater if the additional limb was added. There was only one failure at 1200 N and that the diastasis of the Lisfranc interval was statistically significant for increased widening with the use of IB alone then compared with IB plus additional intercuneiform stability. In this paper, they found that all four failures were female, and that they failed in at least 160% of their body weight. Typical walking produces about 150% of body weight per cycle/load. Therefore, from these studies, it can be deduced that possible early weight-bearing and return-to-work is viable.

In our retrospective review, we identified a total of 15 patients from two level 1 trauma centers. All were fixed in the same technique previously described. From our review, eight of our patients received the standard ligamentous fixation alone, and seven had the additional limb added for intercuneiform instability. Our patients had full return to non-protected weight-bearing at an average of 6.6 weeks (4.1–13.1) and return-to-work/sport at an average of 14.1 weeks (11.6–18) (Table 3). This is an important finding as the time to return-to-work/sport as tolerated was comparable (if not superior) to that of return-to-sport after ORIF with screws and combined bridge plating (19.6 weeks) or suture button (19.4 weeks) previously published [12]. Although we did not truly define the level of participation upon return-to-sport, close to half of our patients were injured in sporting activities (collegiate level) and were allowed to return. Larger studies, especially looking at athletes, would need to be performed to confirm whether this allows quicker return-to-sport, which is outside the scope of this paper.

There were no major complications or hardware failures in this cohort. If, with the InternalBrace, there was fixation failure or loss of reduction, the technique for revision would depend on the specific patient and the mechanism of failure. Hypothetically, if the patient was 65 years or older, had evidence of fixation failure and arthritis, the authors’ preferred treatment would likely be arthrodesis. If the patient was a collegiate athlete under 25 years of age, we would likely consider revision flexible fixation or open reduction internal fixation with screws/plates depending on the specific mechanism of failure.

While the results of this study are very promising, there are of course limitations to our paper. The relatively short term follow-up of this study is a limitation, as arthritis development and long-term fixation failures were not assessed. The small sample size included and the lack of patient reported outcomes were limitations as well. The relatively small sample size of our study did not allow comparison of isolated ligamentous injuries with injuries that also had associated fractures. It is the authors’ opinions that the InternalBrace can still be used when there are small ligamentous avulsions at the base of the second metatarsal and medial cuneiform. The specific nature of each fracture needs to be evaluated by surgeon and larger/comminuted fractures of the Lisfranc joint may prohibit use. Of note, there was one patient in this cohort that had a simple medial cuneiform axial split fixated with independent lag screws, and the InternalBrace was still successfully used to stabilize the Lisfranc joint itself.

In conclusion, our retrospective review from two large academic hospitals demonstrated that for a ligamentous Lisfranc injury, the InternalBrace and additional flexible fixation limb for intercuneiform instability is a reasonable option. Our study demonstrated that with an average of 7-month follow-up, there were zero failures, all persons returned to work/play in less than 15 weeks, and unrestricted weight-bearing began at 6.6 weeks on average. This is the first paper to our knowledge that has reported outcomes of this technique in a non-biomechanical setting. Flexible suture button fixation for Lisfranc injuries continues to be recognized as a viable and stable fixation method [13] but a future prospective, comparative study is necessary to determine if this specific InternalBrace technique is comparable or superior to other techniques.

## Data Availability

The participants of this study did not give written consent for their individual data to be shared publicly. Data stripped of identifying features (gender, age, etc.) are available from the corresponding author, PW, upon reasonable request.
